# Degradation of tetrabromobisphenol A in a paddy soil during sequential anoxic-oxic incubation: Kinetics, metabolites, and potential pathways

**DOI:** 10.1038/s41598-018-31723-9

**Published:** 2018-09-07

**Authors:** Gaoling Wei, Haiqing Zhao, Deyin Huang, Meifang Hou

**Affiliations:** 1 Shanghai Institute of Technology, Shanghai, 201418 China; 2 Guangdong Key Laboratory of Integrated Agro-environmental Pollution Control and Management, Guangdong Institute of Eco-environmental Science & Technology, Guangzhou, 510650 China

## Abstract

Due to the increasing pollution of tetrabromobisphenol A (TBBPA) in paddy soils, it is of great importance to explore the degradation of TBBPA under repeated anoxic-oxic conditions. In the present study, the degradation of TBBPA (kinetics, metabolites and potential pathways) and the influence of low molecular weight organic acid i.e., lactic acid were investigated in a paddy soil during sequential anoxic-oxic incubations. Under the anoxic condition, TBBPA in the non-sterile soils was efficiently debrominated into three intermediates (including tri-BBPA, di-BBPA and mono-BBPA) and bisphenol A (BPA) with a rate constant (*k*) of 0.0371 d^−1^ and a half-life (*t*_1/2_) of 60.8 d. The debromination end product (BPA) steadily accumulated. Next, turning to the oxic conditions, the anaerobically accumulated BPA degraded rapidly, while the intermediates and TBBPA were desorbed from the bound residues and were persistent. The detection of tri-BBPA followed by di-BBPA and mono-BBPA thereafter indicated that the dehalogenation of TBBPA was likely a stepwise removal of bromine atoms. A pathway of TBBPA → tri-BBPA → di-BBPA → mono-BBPA → BPA was thus proposed for TBBPA degradation. The degradation of TBBPA and its metabolites was biologically mediated. Moreover, the biodegradation of TBBPA could be significantly accelerated by the addition of lactic acid as an exogenous carbon source and electron donor, with *k* being increased to 0.0766 d^−1^ and *t*_1/2_ being shortened to 31.9 d. The information will improve our understanding of biotic process associated with agronomic practices (such as applying organic fertilizers) contributing to TBBPA attenuation in the natural soil environment.

## Introduction

Tetrabromobisphenol A [4,4′-isopropylidenebis(2,6-dibromophenol); TBBPA] is one of the most commonly used brominated flame retardants in the world. Given the frequent detection, high persistence and potential toxicity of TBBPA^1,2^, it is of great interest to investigate the dissipation of TBBPA in the environment.

According to previous studies, the reductive dehalogenation is a critical step in the degradation of multi-halogenated compounds^3–5^. The dehalogenated products might be more efficiently degraded by the microorganisms by comparison with their multi-halogenated parent compounds^6^. Because of the significantly faster degradation of the dehalogenated products under oxic conditions compared to anoxic conditions^7^, a multistage process, including both anoxic and oxic stages, may be the best solution for the detoxification of TBBPA in the environment.

Due to the alternation of rainy-dry seasons and various agronomic practices (including flooding and draining, plowing and fallow in winter), the paddy soils are subjected to repeated redox cycles between anoxic and oxic states^8–10^. In view of the reality of increasing TBBPA contamination and repeated redox cycles in the paddy fields, it is of great importance to investigate the biodegradation of TBBPA in soils during the sequential anoxic-oxic process and to develop abatement measures. The biodegradation of halogenated pollutants in soils may be affected by various factors such as the pH, salinity, temperature, availability of organic carbon, mineral composition and microbial community^11–13^. In soils, particularly in iron-rich red paddy soils, ferrous iron plays an important role in the reductive transformation of chlorinated compounds^14,15^. With the participation of soil microorganisms, chlorinated compounds may undergo faster transformation due to both of the reductive ability of the microorganisms and the biogenic Fe(II) formed by the microorganisms^16^. For example, iron-reducing bacteria in soils can reduce iron minerals into biogenic Fe(II) and thereby enhance the dechlorination rate of DDX under anoxic conditions^17,18^. Additionally, applying organic fertilizers containing abundant low molecular weight organic acids usually increases the content of soil organic carbon^19^. Utilized as the carbon and energy source for the microorganisms^20^, the soil organic carbon may have a significant effect on the activities of microbes and consequently affect the biodegradation of halogenated pollutants. Low-molecular-weight organic acids added as exogenous carbon sources have been shown to stimulate the anoxic dechlorination of pentachlorophenol and DDT in soils^4,18,21^. However, the influence of low molecular weight organic acids (e.g., lactic acid) introduced by agronomic practices, such as fertilization, on the degradation of TBBPA in soils is still unclear and needs to be investigated further.

In the present study, the sequential anoxic-oxic incubations of TBBPA were performed in a paddy soil from the Longtang area of South China, a main e-waste recycling area which was extensively polluted by TBBPA and bisphenol A (BPA), supplemented by lactic acid as an exogenous carbon source. The aims are (i) to elucidate the degradation kinetics, metabolites and potential pathways of TBBPA in the paddy soil and (ii) to illustrate the influence of lactic acid on the degradation of TBBPA. The information will improve our understanding of biotic process associated with agronomic practices (such as applying organic fertilizers) contributing to TBBPA attenuation in the natural soil environment.

## Results

### Degradation of TBBPA and formation of its metabolites in a paddy soil

**Figure 1:**
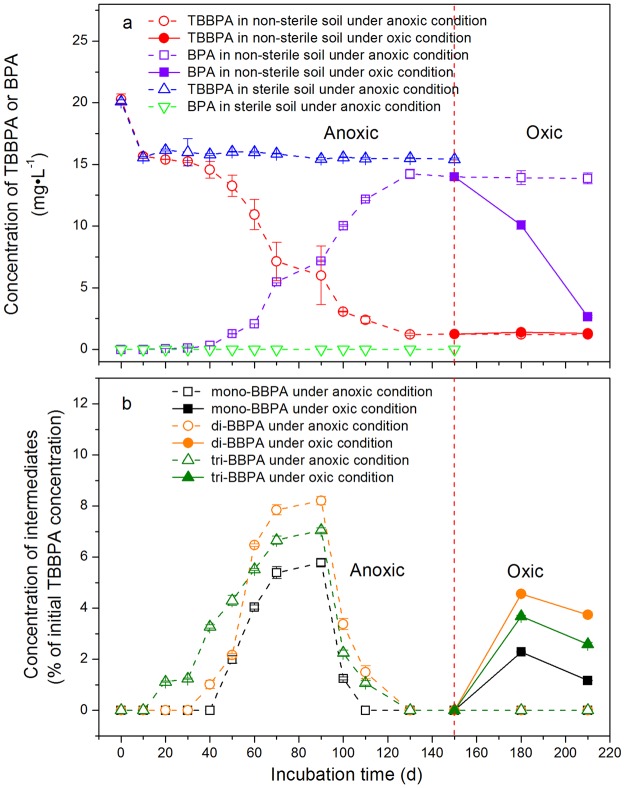
Degradation of TBBPA and formation of metabolites in non-sterile and sterile paddy soil during the incubations of TBBPA under the static anoxic conditions (210 d for non-sterile soil; 150 d for sterile soil) or sequential anoxic-oxic conditions (0–150 d: anoxic, hollow symbols; 151–210 d: oxic, solid symbols). (**a**) The degradation of TBBPA and formation of BPA in non-sterile and sterile soils, respectively. (**b**) The formation of intermediates (mono-, di-, and tri-BBPA) in the non-sterile soil. No metabolites were detectable in the sterile soil. Data based on three replicates were presented as mean ± standard deviation (SD).

In the sterile soils, the concentration of TBBPA was found to be reduced by 22.4% (from 20.1 ± 0.14 to 15.6 ± 0.05 mg·L^−1^), while no metabolites were detectable in the first 10 days of the anoxic incubations. After 10 d, the concentration of TBBPA rose back to 16.2 ± 0.07 mg·L^−1^ and later varied insignificantly from 15.4 ± 0.08 to 16.2 ± 0.07 mg·L^−1^. However, the lower brominated metabolites and BPA were still undetectable throughout the 150 d incubations (Fig. 1a).

In the non-sterile soils, the concentration of TBBPA also dropped by 22.8% (from 20.3 ± 0.41 to 15.7 ± 0.17 mg·L^−1^) during the first 10 days of the anoxic incubations (Fig. 1a). After 10 d, unlike the sterile treatment, the concentration of TBBPA in the non-sterile soils declined continuously, decreased sharply after 50 d and was reduced by 94.0% at 130 d. Correspondingly, the concentration of BPA was 0.06 ± 0.01 mg·L^−1^ at 20 d, increased significantly after 50 d, and maximized at 14.2 ± 0.38 mg·L^−1^ (70.1% of the initially applied TBBPA concentrations) at 130 d. The intermediates were temporarily detectable at 1.07–7.06%, 1.02–8.21% and 1.25–5.78% of the initially applied TBBPA during 20–110 d for the tri-BBPA, di-BBPA, and mono-BBPA, respectively (Fig. 1b). After 130 d, the concentrations of TBBPA and BPA remained stable at 1.22 ± 0.02 mg·L^−1^ and 13.9 ± 0.06 mg·L^−1^, respectively, and the three intermediates were undetectable, leaving BPA as the only measurable degradation product (Fig. 1). The degradation of TBBPA and production of BPA were almost complete at 130 d.

As depicted in Fig. 1b, the tri-BBPA, di-BBPA, and mono-BBPA were first detectable at 20 d, 40 d and 50 d, respectively, simultaneously increased to maxima at 90 d, and decreased significantly and were undetectable after 130 d. During the first 10–50 d, the concentrations of the intermediates were arranged in order of tri-BBPA > di-BBPA > mono-BBPA, and the trend changed to di-BBPA > tri-BBPA > mono-BBPA during 50–130 d (Fig. 1b).

For the non-sterile soil treatments, after the anoxic incubations for 150 d, some of the bottles were exposed to the air and subsequently incubated for 60 d under the oxic conditions. The change of incubation conditions from the anoxic to the oxic state strongly affected the degradation of BPA (Fig. 1a), where the anaerobically accumulated BPA decreased rapidly by 81.1%. However, TBBPA remained stable from 1.24 ± 0.03 to 1.39 ± 0.04 mg·L^−1^ (Fig. 1a). The less brominated intermediates were detected at the ranges of 2.59–3.68%, 3.74–4.56% and 1.17–2.29% of the initially applied TBBPA for tri-BBPA, di-BBPA, and mono-BBPA, respectively. The concentrations of intermediates were in the order of di-BBPA > tri-BBPA > mono-BBPA (Fig. 1b).

### Degradation of TBBPA in a paddy soil with the addition of lactic acid

**Figure 2:**
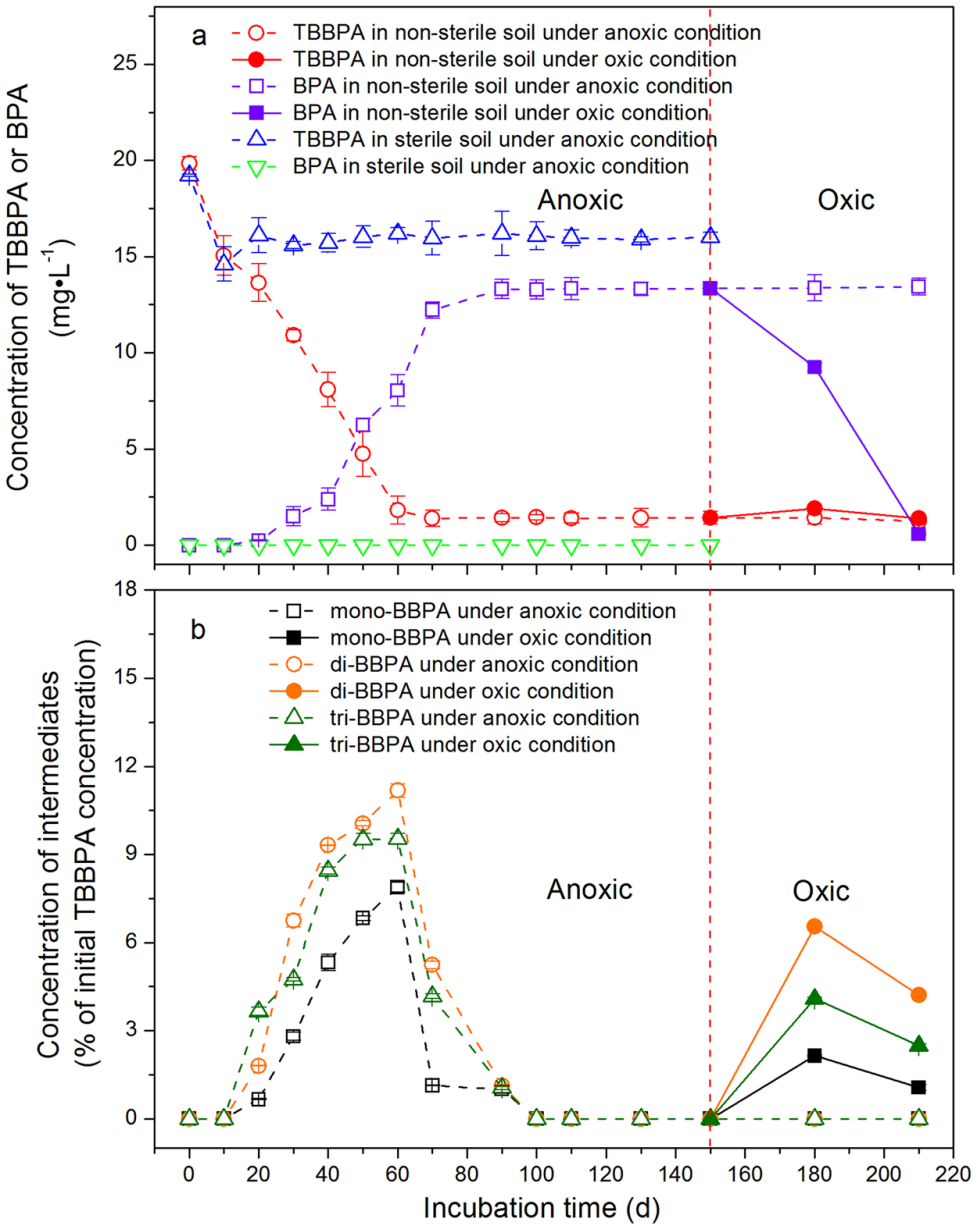
Degradation of TBBPA and production of metabolites in non-sterile and sterile paddy soil with the addition of lactic acid during the incubations of TBBPA under static anoxic conditions (210 d for non-sterile soil; 150 d for sterile soil) or sequential anoxic-oxic conditions (0–150 d: anoxic, hollow symbols; 151–210 d: oxic, solid symbols). (**a**) The degradation of TBBPA and formation of BPA in non-sterile and sterile soils, respectively. (**b**) The formation of intermediates (mono-, di-, and tri-BBPA) in the non-sterile soil. No metabolites were found in the sterile soil. Data based on three replicates were presented as mean ± SD.

Time-dependent profiles of TBBPA and its metabolites in the sterile and non-sterile soils with the exogenous lactic acid addition are depicted in Fig. 2. During the first 10 d of the anoxic incubations, the concentrations of TBBPA decreased both in the sterile (from 19.2 ± 0.05 to 14.6 ± 0.88 mg·L^−1^) and non-sterile (from 19.8 ± 0.36 to 15.1 ± 1.02 mg·L^−1^) soils, with no detection of the debrominated products (Fig. 2a). The reductions of TBBPA in the lactic acid treatments were 24.0% and 24.1% for the sterile and non-sterile soils, respectively.

An insignificant variation of TBBPA (from 15.6 ± 0.18 to 16.2 ± 1.15 mg·L^−1^), as well as no detection of the intermediates and BPA, were observed during 10–150 d of the anoxic incubations in the sterile soils with the addition of lactic acid (Fig. 2a). However, the amount of TBBPA in the non-sterile soils steadily decreased after 10 d, reduced by 93.0% at 70 d, and varied insignificantly from 1.25 ± 0.13 to 1.45 ± 0.13 mg·L^−1^ during 70–210 d. The reductive debromination of TBBPA was almost complete at 70 d. Meanwhile, BPA was first detected at 0.25 ± 0.10 mg·L^−1^ at 20 d, the concentration of BPA increased sharply after 30 d and maximized at 13.3 ± 0.50 mg·L^−1^ (accounting for 67.1% of the initially applied TBBPA) at 90 d, and remained stable from 13.3 ± 0.50 to 13.4 ± 0.45 mg·L^−1^ during 90–210 d. In comparison with the non-sterile treatments without lactic acid, the dissipation of TBBPA and production of BPA were quickened by 60 d and 40 d, respectively, with the addition of lactic acid.

As shown in Fig. 2b, the three intermediates were all detected for the first time at 20 d, increased significantly and reached their maxima at 60 d, then decreased markedly to undetectable levels leaving BPA as the only detectable degradation product after 100 d. The production and disappearance of the intermediates were 30 d early with the addition of lactic acid compared to the lactic acid-free experiment. Tri-BBPA, di-BBPA, and mono-BBPA were individually quantified at 1.05–9.53%, 1.13–11.2% and 0.66–7.88% of the initially applied TBBPA during 20–90 d. The concentrations of the intermediates were initially in the following order of tri-BBPA > di-BBPA > mono-BBPA during 10–25 d, and then changed to di-BBPA > tri-BBPA > mono-BBPA during 25–100 d (Fig. 2b).

For the subsequent oxic incubations of 60 d in the non-sterile soil with the addition of lactic acid, TBBPA varied from 1.40 ± 0.07 to 1.90 ± 0.08 mg·L^−1^, while BPA decreased sharply by 95.7% (from 13.4 ± 0.24 to 0.57 ± 0.05 mg·L^−1^) (Fig. 2a). The lower brominated intermediates were formed again and detected at the ranges of 2.49–4.08%, 4.21–6.55% and 1.06–2.14% of the initially applied TBBPA for tri-BBPA, di-BBPA, and mono-BBPA, respectively. The concentrations of the intermediates were in the order of di-BBPA > tri-BBPA > mono-BBPA (Fig. 2b).

### Fe(II) generation during the anoxic incubations

**Figure 3:**
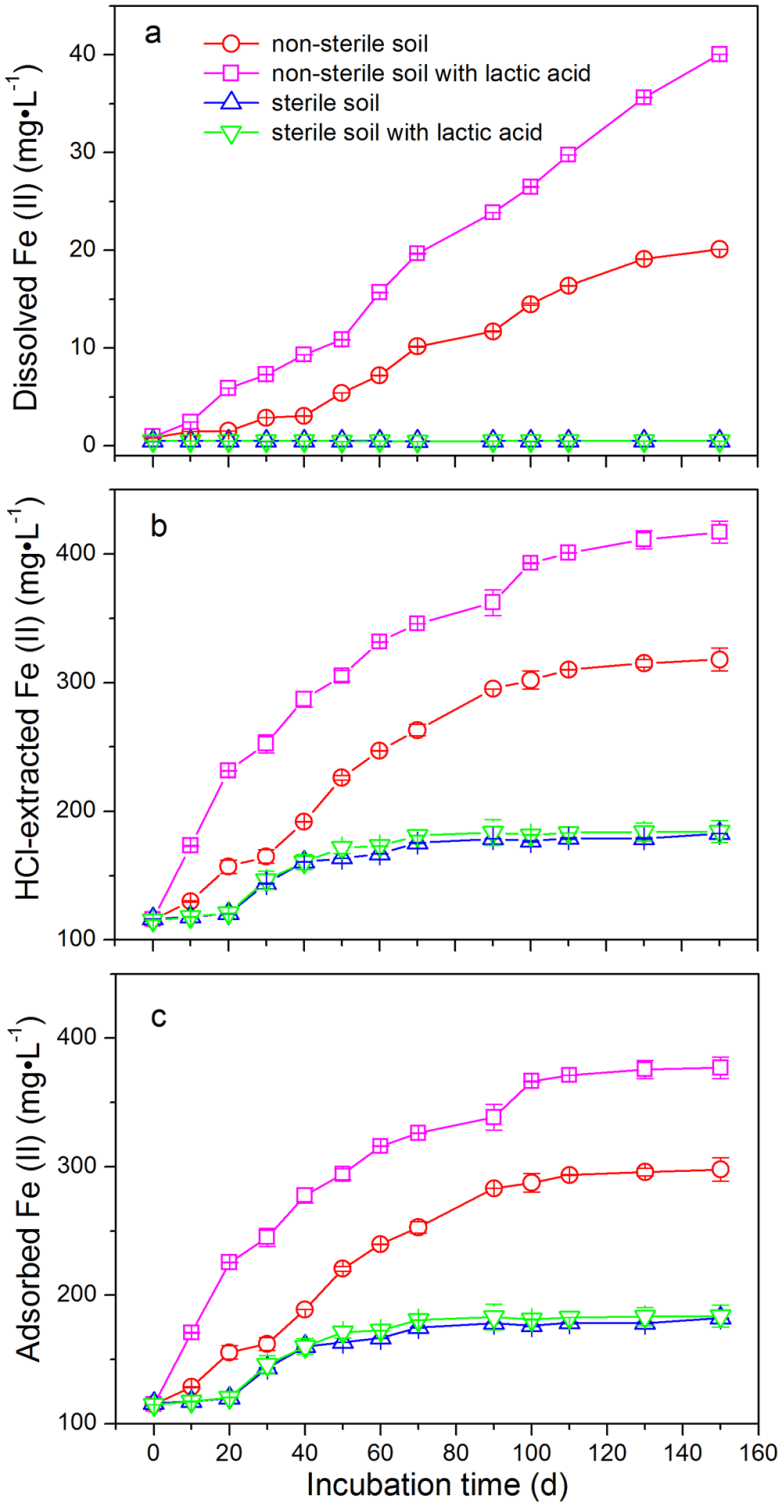
Variations of the amounts of (**a**) dissolved Fe(II), (**b**) HCl-extracted Fe(II) and (**c**) adsorbed Fe(II) during the anoxic incubations of TBBPA in non-sterile and sterile paddy soils with and without the addition of lactic acid, respectively. Data based on three replicates were presented as mean ± SD.

The paddy soil used in the present study is iron-rich soil collected from South China with a high content of iron oxides (31.8 g·kg^−1^). Adsorbed Fe(II) species on mineral surfaces are critical to accelerating the reductive process of organic pollutants^18,22^. Consequently, 0.5 M HCl-extracted Fe(II) has been shown to be effective in extracting produced Fe(II), including adsorbed and dissolved forms^23^. Dissolved Fe(II) species were increased from 0.95 ± 0.002 to 40.0 ± 0.006 mg·L^−1^ and from 0.82 ± 0.001 to 20.1 ± 0.005 mg·L^−1^ in the non-sterile soils with and without lactic acid addition, respectively (Fig. 3a). However, the concentrations of dissolved Fe(II) were stable at ranges of 0.48 ± 0.001–0.51 ± 0.002 mg·L^−1^ and 0.47 ± 0.004–0.51 ± 0.001 mg·L^−1^ for the sterile treatments with and without lactic acid, respectively (Fig. 3a). For the sterile soils, the concentrations of HCl-extracted Fe(II), including the forms of adsorbed and dissolved Fe(II), varied from 115 ± 0.13 to 184 ± 8.42 mg·L^−1^ and from 116 ± 0.08 to 183 ± 0.07 mg·L^−1^ with and without lactic acid, respectively, showing no significant difference (*p* > 0.05) (Fig. 3b). For the non-sterile treatments, the concentrations of HCl-extracted Fe(II) increased markedly and varied from 116 ± 0.13 to 417 ± 8.42 mg·L^−1^ and from 116 ± 0.07 to 318 ± 9.05 mg·L^−1^ with and without lactic acid, respectively. The concentrations of dissolved Fe(II) and HCl-extracted Fe(II) were both significantly higher in the non-sterile treatments compared to the sterile treatments, regardless of whether lactic acid was added (*p* < 0.05). Moreover, the concentrations of dissolved Fe(II) and HCl-extracted Fe(II) in the non-sterile treatments with lactic acid were significantly higher than the concentrations in the treatments without lactic acid (*p* < 0.05). The concentrations of adsorbed Fe(II) under different treatments (Fig. 3c) were in accordance with TBBPA transformation efficiencies. In sterile soils, approximately 160 mg·L^−1^ adsorbed Fe(II) was generated, which was substantially lower than those obtained in the non-sterile soils (Fig. 3c). The highest concentrations of adsorbed Fe(II) were generated in the non-sterile soils with the presence of lactic acid. The results showed significant iron reduction in the non-sterile soils, which indicated that most of the Fe(II) species were microbially generated by the soil microorganisms, where the addition of lactic acid might promote the formation of Fe(II) species during the non-sterile incubations. Thess findings are in good agreement with the above results showing that TBBPA obtained higher transformation rates in the non-sterile soils with the addition of lactic acid.

### Variation of pH values during the anoxic incubations

**Figure 4:**
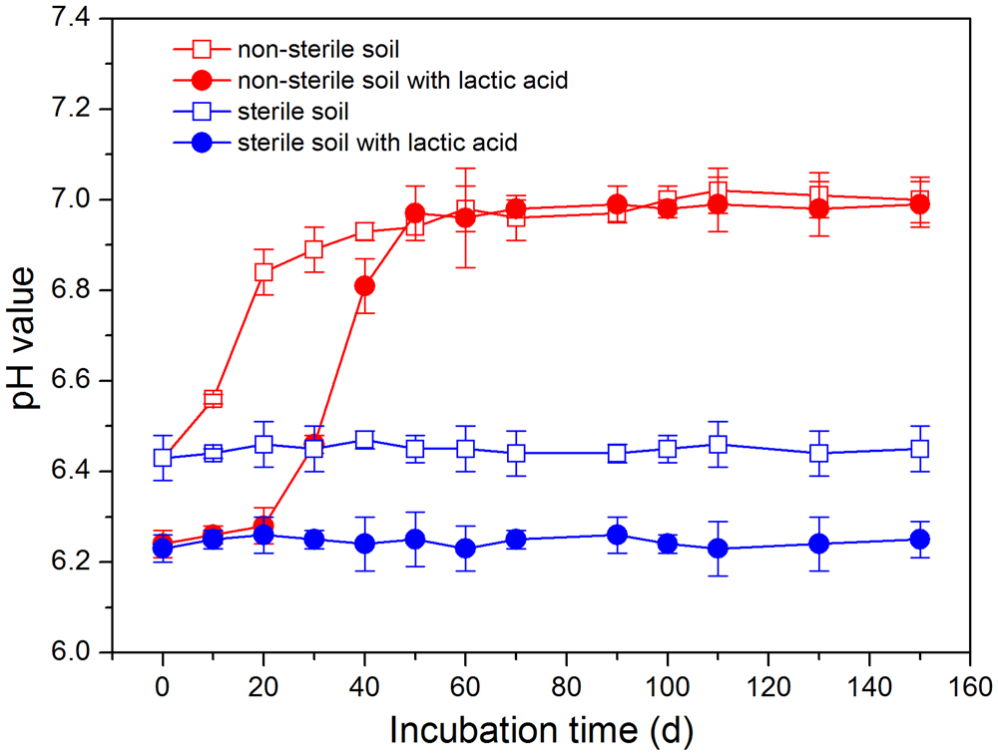
Variations of pH values during the anoxic incubations of TBBPA in non-sterile and sterile paddy soils with and without the addition of lactic acid, respectively. Data based on three replicates were presented as mean ± SD.

During the anoxic incubations in non-sterile soils with and without lactic acid, pH values both slightly increased and varied in a range of 6.09–7.11, exhibiting a little higher in the lactic acid treatments (Fig. 4). It has been documented that TBBPA could biodegrade effectively in neutral and mildly acidic pH media^24^. Apparently, the slightly increased pH values, a result of the dissociation of protein and urea by microorganisms to generate alkaline substances during the anoxic incubations, showed no adverse impact on the microorganisms.

## Discussion

### Degradation dynamics and potential pathway of TBBPA in the paddy soil

As neither BPA nor intermediates of TBBPA were detectable in both sterile and non-sterile soils during the first 10 d, the dissipation of TBBPA could be ascribed to the sequestration of TBBPA in soils, rather than its degradation^25^. No metabolites were found in the sterile treatments throughout the incubations, indicating that TBBPA was not degraded. The increase and insignificant fluctuation of TBBPA after 10 d in the sterile treatments (*p* > 0.05) was likely a consequence of the equilibrium of soil adsorption and desorption. The TBBPA concentration decreased continuously with the concurrent production of the lower brominated intermediates and BPA after 10 d in the non-sterile soils, which suggested that the transformation process of TBBPA was reductive debromination under the anoxic conditions. The reductive debromination of TBBPA was demonstrated to be biologically mediated, which had also been documented previously^3,26,27^.

**Figure 5:**
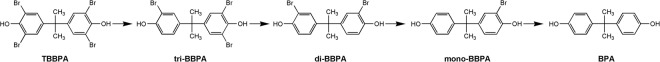
Proposed anoxic biotransformation pathway of TBBPA in the paddy soil.

The detection of tri-BBPA was the earliest followed by di-BBPA and mono-BBPA thereafter, indicating that debromination of TBBPA was probably a stepwise removal of bromine atoms. Therefore, we propose the following biotransformation pathway: TBBPA → tri-BBPA → di-BBPA → mono-BBPA → BPA (Fig. 5). This pathway indicates that the debromination was dominated by the debromination of TBBPA and tri-BBPA during 10–90 d, and then was governed by the debromination of di-BBPA and mono-BBPA during 90–150 d. Both of the OH substituents on the aromatic rings of BPA may be possible sites for degradation, but the two rings joined by a quaternary carbon may inhibit its degradation by anoxic microbes^11^. The steady accumulation of the debromination end product (BPA) during the anoxic incubations confirms that BPA is difficult to degrade. These results provide a good support for the debromination of TBBPA to BPA in anoxic paddy soils and thus explain the levels of BPA higher than TBBPA in paddy soils from the Longtang area^28^. The reductive debromination of TBBPA to the lower brominated intermediates and BPA was also reported in the paddy soils from the Yangtze River Delta^3^.

**Figure 6:**
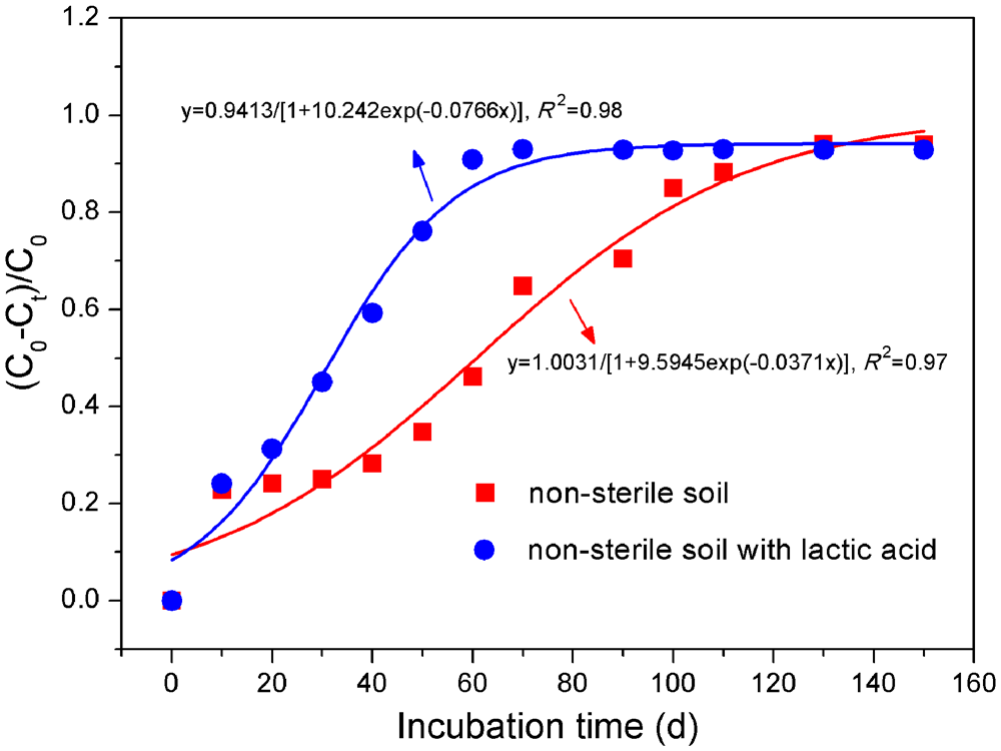
Kinetic curves of TBBPA transformation during the anoxic incubations in non-sterile paddy soils supplemented with and without lactic acid, respectively. *C*_0_ and *C*_t_ were concentrations of TBBPA at the reaction time of 0 and t, respectively.

The rate constant *k* for the reductive debromination of TBBPA in non-sterile lactic acid-free experiments was estimated at 0.0371 d^−1^ (*R*^2^ = 0.97, *p* < 0.05; Fig. 6). Thus, the half-life (*t*_1/2_) was 60.8 d, which was less than the reported degradation half-life of TBBPA under the anoxic conditions in the salt marshes (*t*_1/2_ 70 to >130 d)^29^ and the heavy clay soil (*t*_1/2_ 430 d)^30^ and greater than the reported degradation half-life of TBBPA under the anoxic conditions in the rice paddy soils (*t*_1/2_ 36 d)^3^, the estuarine sediments (*t*_1/2_ 30–40 d)^31^, and the submerged soils planted with reed (*t*_1/2_ 11.4 d) and without any plant growth (*t*_1/2_ 20.8 d)^26^.

The rapid degradation of BPA under the subsequent oxic incubations was because of the existence of BPA-degraders in the non-sterile soils, which was in good accordance with the ubiquitous occurrence of BPA-degrading bacteria in the oxic environments^32^, and the rapid acclimation of the microbial population to degrade BPA^33^. Consequently, the rapid dissipation of BPA in the oxic soils even after long-term anoxic incubations further confirmed the widespread existence and survival of BPA degrading bacteria under different environmental conditions.

The formation of substantial bound residues during the anoxic incubations has been documented, which strongly hinted at the potential release of TBBPA and its metabolites from the bound residues^3,26,34,35^. Considerable amounts of TBBPA and the intermediates were observed during the subsequent oxic incubations, although TBBPA and the intermediates were at a low level (TBBPA) or undetectable (the intermediates) at the end of the preceding anoxic incubations^3^. TBBPA and the intermediates during the subsequent oxic incubations were likely released from the bound residues^3^. The increased levels of TBBPA and the intermediates during the oxic incubations (Fig. 1) showed that no degradation or slower degradation rates compared with the release rates from bound residues. Owing to the formation of bound residues and the release of brominated BPAs, it is strongly recommended the significance of the repeated anoxic-oxic incubations for the complete reductive debromination and mineralization of TBBPA in polluted soils.

### Effects of lactic acid on the transformation of TBBPA in the paddy soil

After adding lactic acid to the sterile soils, there were no detectable metabolites of TBBPA in the sterile soils, which showed that TBBPA did not degrade. However, for the non-sterile soils, TBBPA was debrominated into the lower brominated BPA and BPA, further demonstrating that the microbial activity was the critical factor governing the degradation of TBBPA. The initial dissipation, subsequent increase and insignificant fluctuation of TBBPA in the sterilized soils were in response to the adsorption and desorption of TBBPA to and from soil particles.

With the addition of lactic acid, the degradation in the non-sterile soils was initially dominated by the debromination of TBBPA and tri-BBPA during 10–60 d, and then was governed by the debromination of di-BBPA and mono-BBPA during 60–100 d. Apparently, the reductive transformation of TBBPA and production of BPA, as well as the formation and dissipation of the intermediates, were all accelerated by lactic acid. The transformation of TBBPA was complete at 70 d while the production of BPA was maximum at 90 d (Fig. 2), possibly due to the stimulating effects of lactic acid on the debromination of TBBPA and the lower brominated intermediates. The addition of lactic acid enhanced the TBBPA transformation, with *k* being increased to 0.0766 d^−1^ and *t*_1/2_ being shortened to 31.9 d (*R*^2^ = 0.98, *p* < 0.05; Fig. 6).

The lactic acid, a low-molecular-weight organic acid, can be utilized directly by dehalogenating bacteria as a carbon source^18,36^. Furthermore, the lactic acid can also be decomposed into H_2_, acetate, and propionate by the soil indigenous microorganisms, and the H_2_ that is produced can act as an electron donor for the dehalogenation of the halogenated pollutants. For example, lactic acid has been reported to stimulate dechlorinating microorganisms, and lactic acid might have stimulated the reductive dechlorination of pentachlorophenol in paddy soils^4^. The debromination of TBBPA in the sediments was significantly stimulated by the pyruvate, ethanol, and acetate +H_2_, which might be utilized as electron donors and carbon sources for the microorganisms^37^. In the present study, the addition of lactic acid might stimulate the growth of bacteria that are responsible for the transformation of TBBPA.

**Figure 7:**
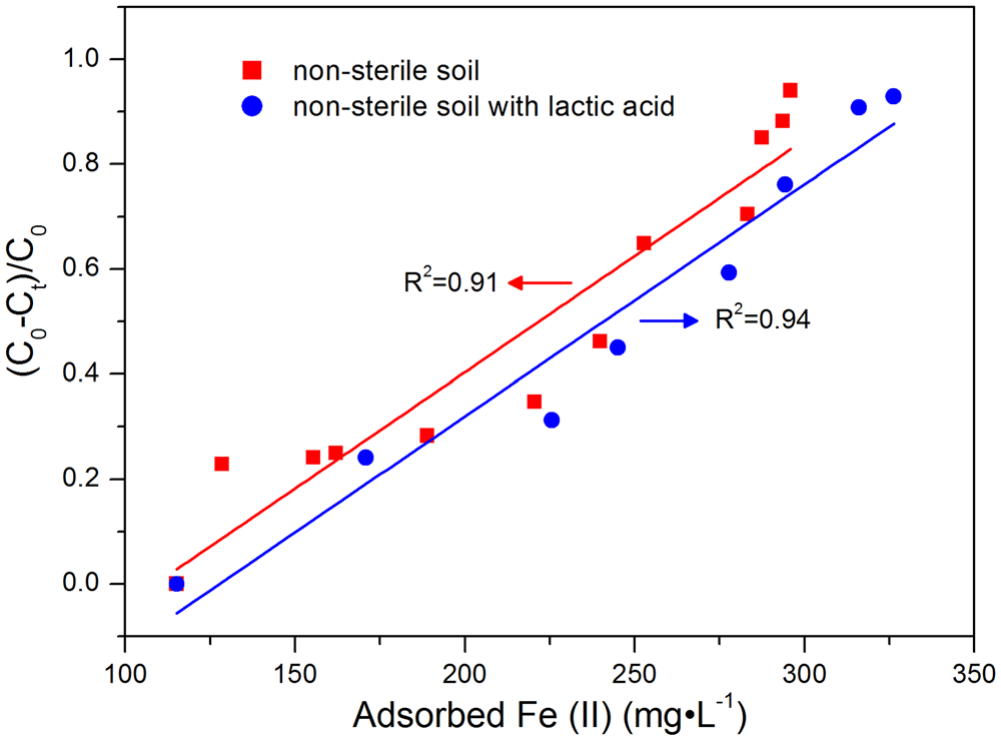
Correlation analysis between the degradation rates of TBBPA and the concentrations adsorbed Fe(II) during the anoxic incubations in non-sterile paddy soils with and without the addition of lactic acid, respectively.

Under the anoxic conditions in soils, the main inorganic electron acceptor Fe(III) could be biologically reduced to Fe(II) by a series of dissimilatory iron-reducing bacteria by coupling the energy generated from the oxidation of organic substrates^38^. The Fe(II) species that are formed have been widely recognized as reducers for reductive degradation of soil pollutants^39^. The reduction of Fe(III) to Fe(II) depends mainly on the availability of organic carbon for microbial growth, where organic acids, such as lactic acid, acetic acid and oxalic acid, can reductively dissolve Fe(III)^40^ and serve as the electron donors for the dissimilatory iron-reducing bacteria during the Fe(III) reduction^38^. Lactic acid has been confirmed to have a stimulated effect on iron-reducing and dechlorinating microorganisms and consequently accelerates the transformation of pentachlorophenol in paddy soils^4^. In the present study, significantly positive correlations were observed between the degradation rates of TBBPA and the concentrations of adsorbed Fe(II) in the non-sterile soils with and without the addition of lactic acid (*R*^2^ = 0.94 and 0.91, respectively, Fig. 7). It is indicated that the adsorbed Fe(II) was an important factor affecting the reductive degradation of TBBPA under anoxic conditions. Moreover, iron reduction in the non-sterile soils is stimulated to form more adsorbed Fe(II) with the addition of lactic acid, and subsequently enhances the reductive debromination of TBBPA.

## Methods

### Chemicals and materials

The standard substances of TBBPA and BPA (purity >98%), and a mixture of N,O-bis(trimethylsilyl)trifluoroacetamide and trimethylchlorosilane (99:1, v-v) were purchased from AccuStandard (New Haven, CT, USA). The solid phase extraction columns (cleanert S C18, 500 mg/6 mL) were purchased from Agela Technologies (Wilmington, NC, USA). The acetone, ethyl acetate and n-hexane (HPLC grade solvents) were from Honeywell International Inc (Morristown, New Jersey, USA). Other chemicals were obtained from the Chemical Reagent Factory (Guangzhou, China).

### Paddy soil

The surface paddy soil used in the present study was sampled from a rice field located in Longtang Town (23°33′39.53′′N, 113°1′59.35′′E), Qingyuan City, Guangdong Province, China. The soil collection method was described previously^28^. The paddy soil was a ferric acrisol containing 3.24% organic matter and 0.20% nitrogen with a pH (0.01 M CaCl_2_) of 6.1 and a cation exchange capacity of 17.6 cmol(+)·kg^−1^. TBBPA, the lower brominated intermediates and BPA were undetectable in the soil. The paddy soil was air-dried, separated from plant residues and stones and later passed through a 100-mesh (0.15 mm) sieve prior to use. Soil for the control experiment was sterilized with high energy γ-rays released from a ^60^Co radioactive isotope and was sealed and stored below −20 °C before use.

### Incubation under the anoxic conditions

Approximately 2 g of soil (dry weight) were placed into a 50 mL serum glass bottle and submerged with 20 mL oxygen-free medium containing NH_4_Cl (2.7 g·L^−1^), MgCl_2_·6H_2_O (0.1 g·L^−1^), CaCl_2_·2H_2_O (0.1 g·L^−1^), FeCl_2_·4H_2_O (0.02 g·L^−1^), K_2_HPO_4_ (0.27 g·L^−1^) and KH_2_PO_4_ (0.35 g·L^−1^). Resazurin was added to three of the bottles as redox indicator at 20 mg·L^−1^. The anoxic incubation treatments included (1) sterile soil + TBBPA; (2) sterile soil + lactic acid + TBBPA; (3) non-sterile soil + TBBPA, and (4) non-sterile soil + lactic acid + TBBPA, where the final concentrations of TBBPA and lactic acid were 20 mg·L^−1^ and 0.1 mmol·L^−1^, respectively. Each treatment was performed in triplicate. After adding the designated reagents, the bottles were purged with N_2_ (99.99%) for 30 min, and sealed with silicone-lined septa and aluminum caps. The serum bottles were placed in the dark in a biochemical incubator at 25 ± 1 °C for incubation for 150 days. At each of the designated sampling times, three bottles were randomly taken for the analysis of dissolved and HCl-extracted Fe(II), TBBPA and its metabolites, respectively.

### Incubation under the oxic conditions

For the non-sterile soil treatments (noted above 3 and 4), after the anoxic incubations for 150 days, several bottles were continuously incubated under anoxic incubations, while the other bottles were transferred into triangular flasks (50 mL) to undergo oxic incubations. The water layer of the mixture was decanted after centrifugation (3000 rpm), and the soil (pellet) was mixed with a stainless spatula and exposed to the air. Next, the flask was closed with a rubber stopper, placed in a shaker at 100 rpm, and kept in a dark room at 25 °C for further incubation. Each treatment was performed in triplicate. At each of the defined sampling times (0, 30 and 60 days), three flasks were randomly taken for the analysis of TBBPA and its metabolites, respectively.

### Analysis of iron species and TBBPA and its metabolites

The dissolved Fe(II) and HCl-extracted Fe(II) were quantified by 1,10-phenanthroline spectrophotometry after direct filtration and extraction of reaction mixtures with 0.5 M HCl for 1.5 h, respectively^41^. The difference between HCl-extracted Fe(II) and dissolved Fe(II) was defined as adsorbed Fe(II)^23^. TBBPA and its products were identified by gas chromatography/maass spectrometry as previously described^28,42,43^. Due to the unavailability of commercial standards for the intermediates, i.e., tri-, di-, and monobromobisphenol A (tri-BBPA, di-BBPA, and mono-BBPA, respectively), their concentrations were estimated from the peak areas of the chromatograms based on the standard curves of TBBPA and BPA on a molar basis with compensation for the number of bromine atoms^43^. Recoveries of the added TBBPA and BPA in the extraction and determination process were 83.4 ± 6.85% and 86.3 ± 8.19%, respectively.

### Data analysis

A kinetics model modified from the microbial logistic growth Eq. (1) has usually been applied to describe the TBBPA transformation process, depicted as Eq. (2)^21,44,45^.

1 $$N({\rm{t}})=A/(1+B{e}^{-kt})$$

2 $$({C}_{0}-{C}_{t})/{C}_{0}=A/(1+B{e}^{-kt})$$

where *A* is the maximal transformation amount of TBBPA, *B* is the regression coefficient, *k* is the rate constant of TBBPA transformation, and *C*_0_ and *C*_t_ are the concentrations of TBBPA at the reaction time of 0 and t, respectively. The half-life time (*t*_1/2_) can be calculated as

3 $${t}_{1/2}=-\,\mathrm{ln}(\frac{A-{C}_{50 \% }}{B\cdot {C}_{50 \% }})\cdot \frac{1}{k}$$

where *C*_50%_ is the concentration of TBBPA when half of the applied TBBPA is gone^46^.
